# Analysis of a Spontaneous Non-Motile and Avirulent Mutant Shows That FliM Is Required for Full Endoflagella Assembly in *Leptospira interrogans*

**DOI:** 10.1371/journal.pone.0152916

**Published:** 2016-04-04

**Authors:** Célia Fontana, Ambroise Lambert, Nadia Benaroudj, David Gasparini, Olivier Gorgette, Nathalie Cachet, Natalia Bomchil, Mathieu Picardeau

**Affiliations:** 1 Institut Pasteur, Unité Biologie des Spirochètes, Paris, France; 2 Laboratoire Lyon Gerland, Merial, Lyon, France; 3 Centre de Recherche Clinique de Saint Vulbas, Merial, Saint Vulbas, France; 4 Institut Pasteur, Ultrapole, Paris, France; University of Kentucky College of Medicine, UNITED STATES

## Abstract

Pathogenic *Leptospira* strains are responsible for leptospirosis, a worldwide emerging zoonotic disease. These spirochetes are unique amongst bacteria because of their corkscrew-like cell morphology and their periplasmic flagella. Motility is reported as an important virulence determinant, probably favoring entry and dissemination of pathogenic *Leptospira* in the host. However, proteins constituting the periplasmic flagella and their role in cell shape, motility and virulence remain poorly described. In this study, we characterized a spontaneous *L*. *interrogans* mutant strain lacking motility, correlated with the loss of the characteristic hook-shaped ends, and virulence in the animal model. Whole genome sequencing allowed the identification of one nucleotide deletion in the *fliM* gene resulting in a premature stop codon, thereby preventing the production of flagellar motor switch protein FliM. Genetic complementation restored cell morphology, motility and virulence comparable to those of wild type cells. Analyses of purified periplasmic flagella revealed a defect in flagella assembly, resulting in shortened flagella compared to the wild type strain. This also correlated with a lower amount of major filament proteins FlaA and FlaB. Altogether, these findings demonstrate that FliM is required for full and correct assembly of the flagella which is essential for motility and virulence.

## Introduction

Leptospirosis is a life-threatening disease representing the most worldwide spread zoonosis, infecting all mammals including humans [1]. This neglected and emerging disease is caused by the spirochete *Leptospira*, a helical bacterium with characteristic hook and spiral shaped ends [2]. Infection with one of the 10 pathogenic species, including *Leptospira interrogans*, results in a broad spectrum of symptoms in humans ranging from subclinical infection to multiple organ failure with a mortality rate of 10 to 50% [3]. Scarcity of efficient genetic tools for the analysis of these slow-growing bacteria has largely hindered our understanding of basic aspects of their biology and virulence mechanisms. Therefore, only a limited number of virulence determinants have been identified so far. They include proteins involved in entry and dissemination in the host, cell adhesion, host tissue damage, adaptation to stress conditions and persistence in the host [4].

The motility of spirochetes is unique in that they can swim in highly viscous gel-like media that classically slow down or stop the motility of other flagellated bacteria [5]. This motility is conferred by two endoflagella inserted subterminally in the periplasm at each pole, extending toward the middle of the cell without overlapping. Whether the cell ends are hook or spiral-shaped is defined by flagella rotation direction. Rotation in a counterclockwise direction (CCW) as seen from the center of the cell forms spiral-shaped ends whereas rotation in a clockwise direction (CW) results in hook-shaped ends. Symmetric rotation of flagella leads to non-translational motility. Translational motility only occurs when flagella are rotating in opposite directions, with the anterior end in helical-shape and the posterior end in hook-shape [2].

Flagella of the model organisms *Escherichia coli* and *Salmonella enterica* have been widely studied (reviewed in [6–10]). The structure of the flagellum, which is composed of a filament, a hook and a motor constituting the basal body, is highly conserved within bacteria [11]. However, the flagellar filaments of spirochetes are not composed of a single flagellin polymer like in enterobacteria, but of a multi-protein complex [12]. In *Leptospira*, site-directed or random mutagenesis allowed the study of some of the components of the endoflagellum, including the filament proteins FlaA and FlaB [13,14], as well as FliY, a flagellar motor protein [15], but the function of these proteins has not been confirmed by genetic complementation.

In this study, we isolated a spontaneous non-motile strain from the pathogen *L*. *interrogans* displaying altered cell morphology and attenuated virulence. Whole genome sequencing of this non-motile strain revealed a single-nucleotide polymorphism in *fliM* resulting in a premature stop codon. Genetic complementation of *fliM* restored motility, cell morphology and virulence. Analysis of purified flagella identified FliM as essential for the synthesis of the full-length endoflagella. We thus identified a flagellar protein essential for hook-shape morphology of the cell ends, assembly of the flagella, motility and virulence in *L*. *interrogans*.

## Material and Methods

### Strains

*Leptospira interrogans* serogroup Australis strains 702 (subsequently called 702^mot-^), 702^compl^ and 733 (also referred to as Wild-Type (WT) cells) were grown in liquid or solid (1% agar plates) Ellinghausen-McCullough-Johnson-Harris (EMJH) at 29°C. *Leptospira* strains were identified at species (16S rRNA sequencing), serogroup (MAT with rabbit antisera) and genotype (MLVA) levels by the French National Reference Center and World Health Organization Collaborating Center for Leptospirosis (Institut Pasteur, Paris, France). Conjugative *Escherichia coli* strains π1, β2163, and BL21(DE3) were cultivated at 37°C in LB as previously described [16]. Spectinomycin and kanamycin, when required, were added to the cultures at a final concentration of 50 μg/ml and 30 μg/ml, respectively.

### Genome sequencing

DNA was extracted with the QiaAmp DNA Blood Midi kit and the DNA Mini kit (Qiagen) according to manufacturer protocols. The genome sequence of strain 702 was determined by Illumina MiSeq v2 2 × 100 nt paired-end at the Genomic Platform of Institut Pasteur (Paris, France). Sequencing reads were assembled using CLC Genomics Workbench 7.0.4 (CLCbio) and annotated with Glimmer logarithm from GenostarSuite 4.0 (Genostar). Genomic sequence polymorphisms between strains 702, 733 and other *L*. *interrogans* strains present in the NCBI database (http://www.ncbi.nlm.nih.gov/nucleotide) were detected and analyzed with GenostarSuite 4.0 and validated by mapping the sequencing reads on identified regions with CLC Genomics Workbench 7.0.4. Single nucleotide polymorphisms (SNPs), insertions and deletions were considered real if these changes were present in at least 70% of reads for a given gene. Nonsynonymous substitutions, insertions or deletions were further confirmed *via* amplification with Phusion High-fidelity DNA polymerase (ThermoFisher Scientific) and sequencing of amplified products.

### Genetic complementation

The coding sequence of *fliM* (LIC11836) was amplified from genomic DNA of *L*. *interrogans* serovar Manilae strain L495 (100% sequence identity with FliM from strain 733 at the amino acid level) using primers FlimF (5′- CGACACATATGACAGAAATTTTAT -3′) and FlimR2 (5′- TAACTTCAATTCTAATATTCTTGTTCAGAACG -3′), containing a restriction site for NdeI (underlined bases), and cloned into pCRII-TOPO vector (Invitrogen) according to manufacturer instructions. The *fliM* coding sequence was then digested with NdeI and XhoI and inserted into the same restriction sites of pPflgB plasmid [12] to generate a transcriptional fusion with the *Borrelia burgdorferi flgB* promoter. The P*flgB*-*fliM* DNA fragment was released by EcoRI digestion and cloned into the corresponding site of the replicative plasmid pMaORI [17] to generate pCF036. The *fliM* plasmid construct was introduced in strain 702^mot-^ by conjugation with *E*. *coli* β2163 carrying pCF036 as previously described [17]. The transformed bacteria were plated onto EMJH plates containing 50 μg/ml of spectinomycin and incubated at 29°C for 5 weeks. Colonies were then inoculated into liquid EMJH supplemented in spectinomycin for further analysis.

### Motility assays

Mid-log cultures of leptospires were observed by dark-field microscopy with or without 1% methylcellulose (Sigma), using ×20 to ×200 magnification with an Olympus BX-53 139 microscope connected to a Hamamatsu Orca Flash 2.8 camera. Images were taken with the CellSense Software (Olympus) and videos were recorded with the MicroManager 1.4 software (μManager). Images were post-treated and analyzed with the ImageJ software (ImageJ). Three images of each strain containing approximately 90 bacteria were used to measure cell length. Motility of *Leptospira* strains was also evaluated in triplicate onto 0.5% agar EMJH plates by inoculating 5 μl of mid-log phase cultures (OD_450nm_ of approximately 0.3). Plates were incubated at 29°C for 10 days.

### Recombinant FliM purification and antisera raising

The *fliM* coding sequence was cloned into the NdeI and BamHI sites of the pET28 vector (Novagen). The resulting plasmid, which allowed expression of a N-terminal (His)6-tagged recombinant FliM, was introduced into *E*. *coli* BL21(DE3) cells. Recombinant protein expression was induced on 2 L of transformed cells at an OD_600nm_ of 0.7 by addition of 1 mM Isopropyl β-D-1-thiogalactopyranoside (IPTG) for 4 h at 37°C. FliM was highly insoluble in the growth condition used and was purified from inclusion bodies by resuspending cell pellets in buffer A (50 mM NaH_2_PO_4_ pH 8.0, 300 mM NaCl, 10 mM imidazole, 10% glycerol) and sonication in the presence of protease inhibitor cocktail (complete mini EDTA-free, Roche) and 1 mM phenylmetylsulfonyl fluoride (PMSF). The insoluble fraction obtained after a 60 min centrifugation at 40,000 × *g* at 4°C was resuspended in buffer A containing 1% Triton X100 to solubilize membrane proteins. Inclusion bodies obtained after a 30 min centrifugation at 40,000 × *g* were solubilized in buffer B (100 mM NaH_2_PO_4_ pH 8.0, 8 M urea, 10 mM Tris pH 8.0, 200 mM NaCl, 1 mM PMSF, 0.1% Triton X100), incubated for 1 h at room temperature with stirring and centrifuged for 30 min at 4°C at 40,000 × *g*. The supernatant was loaded on a nickel-nitrilotriacetic resin (Ni-NTA, Qiagen) equilibrated with buffer B. Since recombinant (His)_6_-FliM could not be eluted from the column by conventional methods, the flow-through that contained unbound (His)_6_-FliM was used to further purify the protein by filtration on a 100 kDa cut-off cellulose membrane (Amicon Ultra 100K, Merck Millipores). The flow-through after filtration was dialyzed against buffer C (50 mM Tris pH 8.0, 150 mM NaCl, 2 M urea, 2 mM DTT, 5 mM EDTA) and concentrated to 2.5 mg/mL.

To raise polyclonal sera, two rabbits were immunized four times with 2 mg of purified FliM protein, and sera were collected on day 38 (Aldevron, Freiburg, Germany).

### Preparation of periplasmic flagella

Flagella extraction protocol was adapted from Lambert and coworkers [14]. Three independent mid-log phase cultures (approximately 5 × 10^8^ cells/ml) of 200 ml of *Leptospira* strains were centrifuged at 8,000 × *g* for 20 min at 4°C. Pellets were washed with 22 ml PBS and with 24 ml sucrose solution (0.5 M sucrose, 0.15 M Tris pH 8). Cells were resuspended in 12 ml sucrose solution and stirred on ice for 10 min. Triton X-100 was added at 1% final concentration and the mixture was incubated for 30 min at room temperature. 0.1 mg of lysozyme was added and incubated 5 min on ice. Then, EDTA pH 8 was added to a final concentration of 2 mM. After 2 h incubation at room temperature, MgSO_4_ was added to a final concentration of 2 mM and stirred 5 min at room temperature, then EDTA was added to a final concentration of 2 mM and stirred again 5 min at room temperature. The mixtures were then centrifuged at 17,000 × *g* for 15 min at 4°C. The supernatants were mixed with 1.6 ml of 20% polyethylene glycol 8,000 previously dissolved in 1 M NaCl, and incubated for 30 min on ice. Suspensions were centrifuged again at 25,000 × *g* for 45 min at 4°C. Pellets were resuspended in 2.5 ml of water and 100 μl of 1% sodium azide in PBS, and placed at 4°C overnight. The mixtures were then ultracentrifuged 45 min at 80,000 x *g* at 4°C in a Beckman Optima MAX Ultracentrifuge, with rotor TLA 100.3. Flagellar preparations were resuspended in an appropriate volume of water depending on the OD of the culture (approximately 800 μl) and 100 μl of 1% sodium azide.

### Immunofluorescence (IFA)

The protocol was adapted from Pinne and Haake [18]. 5 × 10^8^ leptospires were washed in PBS - 5mM MgCl_2_ and air-dried on 4 wells Lab-Tek® II CC² slides (Nunc) for 1 h at 29°C. The cells were then fixed and permeabilized with 1 ml ice-cold 100% methanol for 20 min at -20°C. Non-specific sites were blocked with 1 ml of PBS-BSA 2% for 1 h. 200 μl of FlaB (LIC11531) primary antibody diluted at 1:100 in PBS-BSA 2% were added, incubated for 1 h and washed 3 times with 1 ml of PBS. 200 μl of secondary antibody Cy^TM^3-conjugated AffiniPure Goat Anti-Rabbit IgG (H+L, Jackson ImmunoResearch) diluted at 1:100 in PBS-BSA 2% were added and incubated for 1 h. Cells were washed twice with 1 ml PBS and once with 1 ml of water. The chambers were removed and the slides were air-dried for several minutes. A drop of ProLong® Gold Antifade reagent containing 4′,6′-diamidino-2-phenylindole (DAPI, Life Technologies) for DNA counterstaining was added and the slides were covered with a coverslip. After an overnight incubation in the dark, the slides were observed with a Nikon Eclipse Ti-S fluorescence microscope (Nikon) coupled to a Digital Sigth DS-5Mc camera (Nikon). 10 images containing approximately 20 flagella were acquired for each strain with the Nikon NIS Elements F Package 4.0 (Nikon) and post-treated with ImageJ.

### Transmission electron microscopy (TEM)

Transmission electron microscopy was performed at the UltraPole Platform at the Institut Pasteur (Paris). Commercial 400 mesh carbon-coated copper grids were glow-discharged just before use. The samples were placed in contact with the grids for 10 min. The grids with samples were then fixed with 2% glutaraldehyde in 0.1 M cacodylate sodium buffer for 10 min and washed 3 times for 1 min in water for biosecurity requirement. The grids were dried and negatively stained with 4% uranyl acetate for few seconds. The samples were observed with a transmission electron microscope (TEM) TECNAI T12 FEI operating at 120 kV. For manual length measurements of flagella, 15 fields containing approximately 25 flagella each were chosen randomly and all flagella were measured using ImageJ.

### Protein electrophoresis and immunoblot

Cultures in triplicate of mid-log phase leptospires (approximately 5 × 10^8^ cells/ml) were centrifuged for 10 min at 7,000 × *g*, washed once in PBS and then normalized in PBS at 1 or 5 OD_450nm_ unit per milliliter. Washed cells and flagellar extracts were mixed with 3X loading buffer (0.2 M Tris pH8, 16% 2-mercaptoethanol, 6% SDS, 30% glycerol and 0.06% bromophenol blue) and boiled at 100°C for 10 min. 4–20% SDS-PAGE gel were loaded with 0.006 or 0.03 OD_450nm_ units of cell lysates for LipL41 and FliM detection, respectively, and 10 μl of flagellar extracts. Western Blots were performed by transferring the proteins from the gels to nitrocellulose membranes. After blocking the membranes with Odyssey buffer (Li-Cor), rabbit antibodies anti-FlaB (LIC11531, 1:1,000), FlaA2 (1:1,000), FliM (1:100) and LipL41 (1:100) were added. Primary antibodies were validated by immunoblot against the corresponding recombinant proteins. Secondary detection was performed with anti-rabbit IgG (H&L) goat antibody IRdye800® Conjugated (Rockland) at a 1:10,000 dilution and Odyssey® imaging system at 800 nm. Measurement of bands intensities with Odyssey® software (Li-Cor) allowed the approximate relative quantification of proteins.

### Virulence assay

Six weeks old female hamsters were purchased from Janvier labs (Janvier, Le Geneste-Saint-Isle, France). The hamsters were housed in ventilated cages (5 hamsters per cage) placed in a temperature-controlled room, with exposure to light 12 hours/day (6:00 a.m.-6:00 p.m.) and fed with adequate autoclaved food and water. Animals were infected with leptospires by intraperitoneal route. One group of 5 hamsters received 10^8^ leptospires of strain 702^mot-^, two groups of 5 hamsters received 10^8^ or 10^6^ leptospires of strain WT and two groups of 10 hamsters received 10^8^ or 10^6^ leptospires of strain 702^compl^. Animals were observed on a daily basis for 21 days. Moribund hamsters were euthanized. The animal experimentation was performed in compliance with the 2010/63/UE directives on the protection of animals used for scientific purposes and the transposed corresponding French decrees and with “Merial Position Statement—Animal Welfare (Animal Testing)”.

### Accession number

The whole genome shotgun project of *L*. *interrogans* strains 702^mot-^ and 733 (WT) has been deposited at DDBJ/EMBL/GenBank under the accession numbers NZ_LMXF00000000.1 and NZ_LMXK00000000.1, respectively.

## Results

### Isolation of a spontaneous non-motile mutant

*L*. *interrogans* serogroup Australis strain 702 was isolated from the blood of a dog with acute leptospirosis. The culture appeared clonal with non-translating leptospires under dark-field microscopy. In addition, only small colonies were obtained by plating the isolate onto solid EMJH media. Assuming that this non-motile phenotype arose from a motile parental strain, we selected the closest phylogenetic strain from our existing collection for further phenotypic analysis. The motile strain 733, which was also isolated from a sick dog visiting the same clinic three months later, exhibited the same genotype (as determined by 16S rRNA and MLVA analyses) and serological features (serogroup Australis) than the non-motile strain 702. Furthermore, growth kinetics in liquid EMJH medium were similar for both strains as measured by absorbance (S1 Fig). Thus, strain 733 can be considered as a WT strain to be compared with strain 702 for following experiments. Strain 733 will consequently be referred to as WT strain.

**Fig 1:**
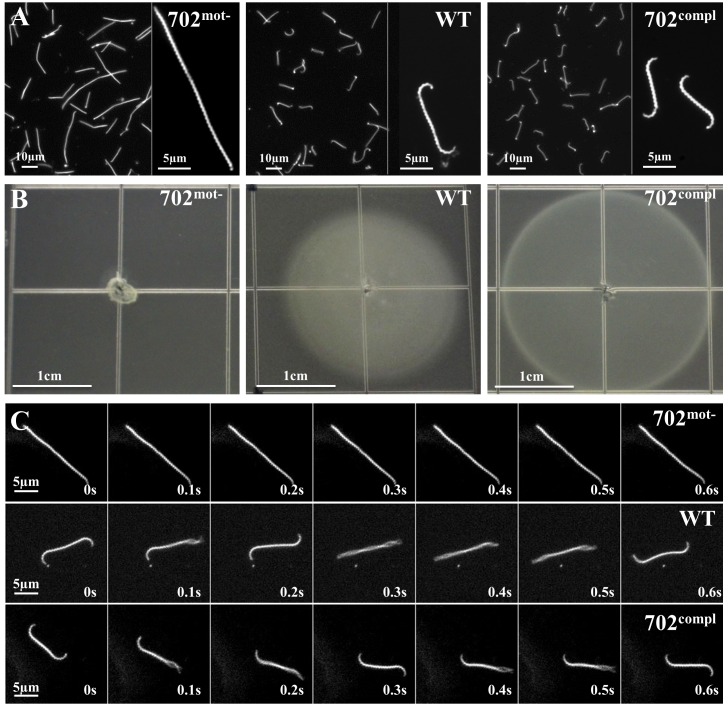
Morphology and motility of 702^mot-^, WT and 702^compl^ strains. (A) Observation under dark-field microscopy at ×20 or ×200 magnifications of cultures in liquid EMJH. (B) Spread of bacteria on soft 0.5% agar EMJH plates observed after 10 days of incubation. Identical results were obtained on 0.3% agar plates (S2 Fig). (C) Images taken every 0.1 s during 0.6 s under dark-field microscopy at ×200 magnification.

Strain 702, further named 702^mot-^, presented an atypical straight cell morphology under dark field microscope, lacking characteristic hook and spiral-shaped extremities, with no translational motility (Fig 1A and 1C, S1 and S2 Movies). Approximately 2% of leptospires showed a small hook at one extremity, conferring sporadic and slow rotation movements unable to propel the cells. We confirmed this absence of motility on soft agar plates, as the strain did not spread from its inoculation site contrary to WT cells (Fig 1B). In addition, this strain showed a filamentous morphology. Average length of WT and 702^mot-^ cells were 9.4 μm and 16.5 μm long, respectively (Table 1). An analysis of the distribution of size of cells from mid-log cultures showed that 99% of the WT cells were shorter than 15 μm and that 1% had a length comprised between 15 μm and 30 μm. By contrast, only 51% of the 702^mot-^ cells were shorter than 15 μm and 43% of the cells had a length between 15 μm and 30 μm; 6% were also longer than 30 μm (S3 Fig). These data indicate probable cell separation defects in strain 702^mot-^.

**Table 1:** Mean cell and flagella length as determined by dark-field microscopy, immunofluorescence (IFA) and electron microscopy (TEM).

	702^mot-^	WT	702^compl^
Cell length (μm) (SD) *	16.5 (8.1)	9.4 (3.2)	10.9 (2.3)
Flagella length by IFA (μm) (SD) **	1.3 (1.1)	3.5 (0.9)	3.6 (0.9)
Flagella length by TEM (μm) (SD) ***	0.9 (0.6)	2.7 (1.3)	1.7 (1.1)

* on 275 cells

** on 210 stained flagella

***on 360 purified flagella (SD) standard deviation

### A single spontaneous mutation abolishes FliM protein expression in the non-motile strain

**Fig 2:**
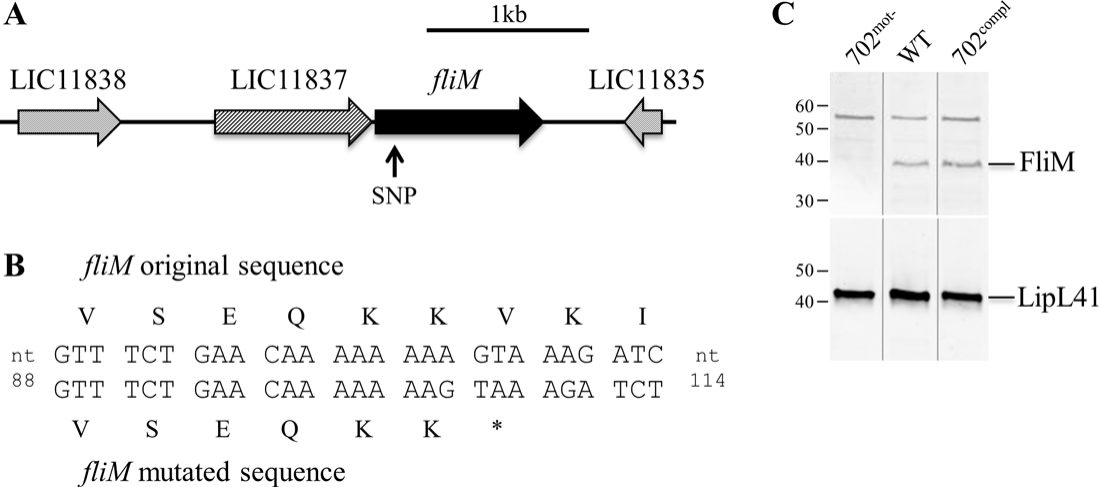
Mutation in *fliM* gene in 702^mot-^ strain. (A) Genetic locus of *fliM* gene in *L*. *interrogans*. The arrow indicates the localization of the SNP in the 702^mot-^ strain. Gene nomenclature corresponds to the homologs in *L*. *interrogans* serovar Copenhageni strain Fiocruz L1-130. (B) *fliM* nucleotide and corresponding amino acid sequences in mutated and WT strains. The deletion at position 105 of the coding sequence in strain 702^mot-^ induces a stop codon. (C) Detection of FliM and LipL41 by immunoblots. Uncropped gels are presented in S4 Fig.

The whole genome of strain 702^mot-^ was sequenced and compared to other available genomes to identify mutation(s) responsible for the motility-deficient phenotype. 50,272,184 reads were analyzed, representing a depth of coverage of 1,000x, assembled into contigs and annotated. Comparative analysis of 702^mot-^ genome sequence with the genomes of *L*. *interrogans* from the NCBI database and strain 733 (WT) revealed only one deletion/insertion polymorphism (DIP) in LIC11836 and one nonsense single nucleotide polymorphism (SNP) in LIC10453. Both mutations introduced premature stop codons. LIC10453 encodes a hypothetical protein conserved among pathogenic *L*. *interrogans* but less conserved in saprophyte *L*. *biflexa* (99% and 62% identity, respectively). LIC11836 is annotated as the flagellar motor switch protein FliM which is highly conserved among pathogenic and saprophytic *Leptospira* spp. (99% and 88% identity in *L*. *interrogans* and *L*. *biflexa*, respectively). The effect of such a mutation was then investigated. The premature stop codon in *fliM* resulted from a single nucleotide deletion 105 nt downstream the start codon (Fig 2A and 2B). Amplification and sequencing of the coding sequence of *fliM* in WT compared to 702^mot-^ confirmed the presence of the SNP only in the non-motile strain. Whole cell content analysis by immunoblot indicated that FliM is produced by the WT strain but not by the 702^mot-^ strain (Fig 2C). Equal loading of the strains in the wells was validated with antiserum against LipL41, whose expression is described as stable in *Leptospira* spp. [19–23].

### Complementation of 702^mot-^ mutant with *fliM* restores cell shape and motility

To confirm that the spontaneous mutation in *fliM* specifically resulted in the loss of motility and altered cell morphology, we complemented the 702^mot-^ strain in *trans* with the replicative vector pMaORI containing a functional *fliM* gene under the control of a constitutive promoter. Because *fliM* is the second gene of a putative operon (Fig 2A), the native *fliM* promoter could not be used for complementation. Expression of *fliM* was restored in the resulting strain 702^compl^ as observed by Western Blot (Fig 2C). Wild type morphology with hook and spiral-ends and regular cell lentght was also restored as observed by microscopy (Fig 1A, Table 1 and S3 Fig). In addition, motility was restored as seen under the dark-field microscope (Fig 1C, S3 Movie) and according to the spread of bacteria on soft agar plates (Fig 1B).

Taken together, these data prove that *trans* complementation successfully reintroduced a functional FliM in strain 702^mot-^ and that FliM is critical for cell shape and motility.

### Motility is essential for virulence

**Fig 3:**
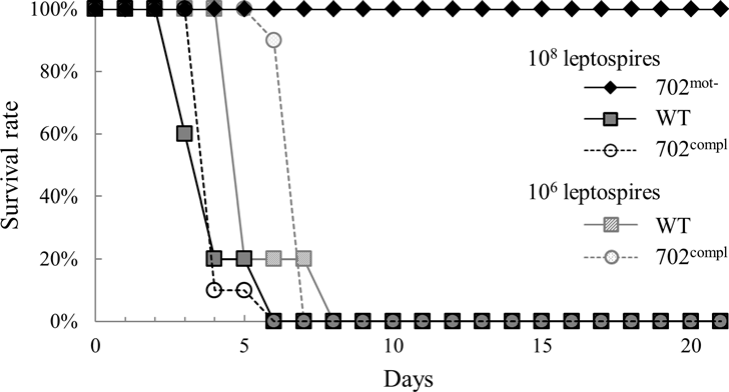
Virulence of 702^mot-^, WT and 702^compl^ strains. The hamsters were infected intraperitoneally with the three strains. One group of 5 hamsters received 10^8^ leptospires of 702^mot-^ strain, 2 groups of 5 hamsters received 10^8^ or 10^6^ leptospires of WT strain and 2 groups of 10 hamsters received 10^8^ or 10^6^ leptospires of 702^compl^ strain.

To investigate the importance of motility in *Leptospira* virulence, groups of hamsters were infected with the 3 strains by intraperitoneal route. All hamsters survived an infection of 10^8^ leptospires with strain 702^mot-^ whereas those infected by WT and 702^compl^ strains at doses of 10^6^ or 10^8^ leptospires died between 6 and 8 days (Fig 3). These data indicate that the non-motile 702^mot-^ is not virulent even at high infection dose and that restoration of motility upon reintroducing a functional FliM correlates with restoration of virulence.

### *fliM* disruption induces a defect in flagella assembly

**Fig 4:**
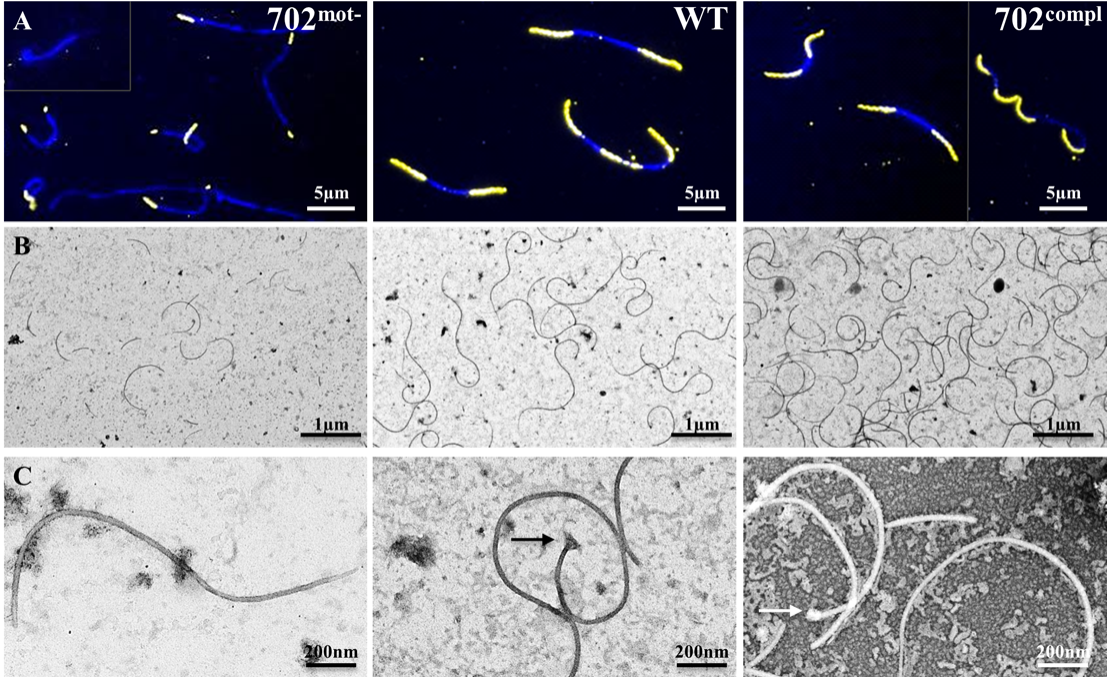
Flagella integrity in 702^mot-^, WT and 702^compl^ strains. (A) Immunofluorescence assay using FlaB antibody on methanol permeabilized cells detected by Cy3 coupled secondary antibodies (yellow) and DAPI counterstaining (blue). Merged images are presented. (B and C) Observation of flagella preparations by transmission electron microscopy after negative staining with uranyl acetate at ×4,800 magnification (B) and ×30,000 magnification (C). The arrows point flagellar motors.

Since FliM is presumably one of the components of the flagellar motor, we assessed the integrity of the flagella in the 702^mot-^ strain. Immunofluorescence assays (IFA) were performed using antibodies directed against the major filament protein FlaB (Fig 4A). Staining of the WT strain revealed the presence of the flagella from the extremities to the center of the cells without overlapping. These results are consistent with previous IFA experiments and descriptions of the endoflagella in *Leptospira* [14]. The staining of the complemented strain 702^compl^ was similar to WT. FlaB stained flagella measured approximately 3.5 μm in these two strains. In contrast, 702^mot-^ showed either shorter flagella of approximately 1.3 μm or no flagella in 30% of the cells (Table 1, S5 Fig). In order to better characterize flagellar length, flagella of the three strains were purified and observed by electron microscopy (Fig 4B and 4C). For WT strains, intact coiled flagella of a median size of 3.2 μm in length were observed (Table 1, S6 Fig), consistent with the IFA experiment. Most of the flagella were composed of the basal body at one extremity and a filament displaying a thinner diameter at the other extremity, probably due to the lack of sheath as already described in previous leptospiral flagella observations [14]. In 702^mot-^, all flagella were significantly shorter, with a median length of 0.7 μm. This observation was confirmed on batches of flagella extracted from three independent cultures. Surprisingly, the length of flagella from 702^compl^ were composed of typical long flagella mixed with short flagella, which is not in agreement with the results obtained by IFA. This may indicate an increased fragility of flagella in the complemented strain, resulting in more broken flagella during the purification of filaments.

**Fig 5:**
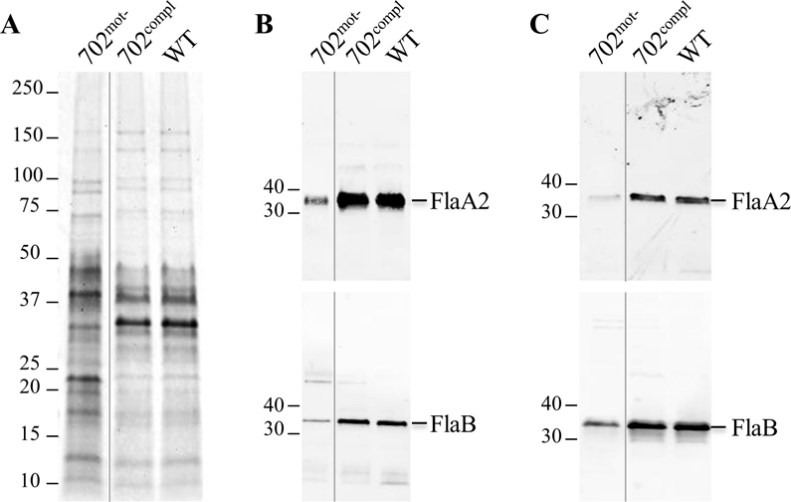
Flagellar protein content in 702^mot-^, 702^compl^ and WT strains. (A) Purified flagella analyzed by Coomassie stained SDS-PAGE. (B and C) Detection of FlaA2 and FlaB by immunoblots in (B) purified flagella and (C) whole cell lysates. Uncropped gels are presented in S7 Fig.

To further characterize the flagella at the protein level, purified flagella were analyzed by electrophoresis. Coomassie staining revealed a different protein content in 702^mot-^ in comparison to WT and complemented strains (Fig 5A). In particular, the amount of major bands corresponding to proteins with an apparent molecular mass of about 30–40 kDa, matching the size of previously described flagellar proteins [14], was lower in 702^mot-^. Moreover, an immunoblot revealed a decreased amount of approximately 2.5 fold of two major filament proteins, FlaA2 (LIC10787) and FlaB (LIC11531), in 702^mot-^ compared to WT and 702^compl^ (Fig 5B). A similar decreased expression was also observed in whole cell lysates (Fig 5C). However, an RT-PCR experiment showed that the transcription of these genes, in addition to other flagellar genes, was not affected in 702^mot-^ strain (S8 Fig). Altogether, these data show that *fliM* disruption leads to a reduced amount of flagellar proteins, correlated with incomplete flagella assembly, and that these short flagella are not able to confer motility.

## Discussion

Motility in leptospires is conferred by the asymmetrical rotation of two periplasmic flagella attached at each extremity of the cell. In this study, we isolated a spontaneous *L*. *interrogans* mutant from a dog with leptospirosis. This strain is avirulent, non-motile and does not exhibit full-length flagella. Genome sequencing and complementation revealed that a single nucleotide deletion in the *fliM* gene, which causes apparition of a premature stop codon, is responsible for this phenotype.

Infection experiments revealed a lack of pathogenicity of the non-motile strain and restoration of full virulence by reintroducing a wild-type *fliM*. It demonstrates that FliM and motility are crucial for virulence. Motility genes, previously shown as highly expressed *in vivo* [24,25], are certainly critical for the leptospires to breach the mucosal membranes, enter the tissues though damaged skin, disseminate in the host and penetrate the organs [4].

This study raises the question of how a non-virulent strain was isolated from a dog who died of leptospirosis. We hypothesize that the point mutation may have occurred following isolation, with the mutated non-motile strain outgrowing the WT population *in vitro*. This is most likely due to a selective advantage, since motility represents significant energy costs without being essential for *in vitro* growth [26]. In *Pseudomonas putida*, it was shown that a non-flagellated strain had advantages compared to the motile WT strain in term of growth, adaptation and resistance to stresses [26]. At this point, we cannot exclude that the dog was co-infected by both a virulent and a non-virulent strain with subsequent enrichment of the population of the non-motile variant in the bloodstream.

Disruption of FliM induced an atypical cell morphology in 702^mot-^ strain. Cells were straight, lacking hook and spiral shaped ends but retained their helical cell body. A similar phenotype was previously reported in non-motile *Leptospira* strains with mutations in flagellar genes *flaA2*, *flaB1* [13,14] or non-identified gene(s) [27–29], supporting the involvement of flagella in the hook and spiral shape of the cell extremities. It has been hypothesized that this may be due to the flagella being more rigid than the cell cylinder and therefore drawing extremities toward the middle of the cell [8]. The *fliM*-deficient strain also showed cell separation defects, with multiple bacteria often attached in chains. The same phenotype was described for the above mentioned non-motile *Leptospira* mutants and in other spirochetes like *B*. *burgdorferi fliG2*, *flaB*, *fliI* and *fliH* mutants [30–32]. It is likely that movements of cell extremities may favor separation of daughter cells during the cell division process but the exact mechanism is currently unknown.

In model bacteria, FliM is described as an important flagellar protein as it regulates rotation and switching of flagellar motor between CW and CCW direction in response to chemotaxis, by directly interacting with signaling protein CheY [2,33–35]. FliM is located in the C-ring, a structure of the basal body composed of additional proteins FliN, FliG and FliY. Absence of FliM probably abolishes interactions with chemotaxis proteins and flagellar motor direction switch, leading to motility deficient strains. This is the case in *Helicobacter pylori* mutants lacking any of the four switch proteins [36], and in spirochetes *B*. *hermsii*, *B*. *burgdorferi* and *L*. *interrogans* in mutant strains lacking *fliG*, *fliH* and *fliY* mutants, respectively [15,32,37].

In *Leptospira*, the function of FliM had never been experimentally described so far. Our findings show that FliM plays a critical role in flagella assembly. In the *fliM*-deficient strain, flagella were either absent or present in a truncated form at one or both extremities, whereas the complemented strain showed full-length and functional flagella. This is consistent with experimental data on *S*. *typhimurium* showing that in the absence of the C-ring, flagella were not formed beyond the MS ring, a basal part of the motor, and that N-terminal and C-terminal parts of FliM were essential for whole flagellar structure assembly [38,39].

Whereas transcription of genes of the flagella seemed not to be altered, the amount of different flagellar proteins, in particular FlaA2 and FlaB, was markedly decreased in the *fliM*-deficient strain. We hypothesize that flagellar proteins may be correctly produced but not properly assembled, thereby further degraded. FliM proteins, quantified by a mass spectrometry based study at 96 copies per cell, corresponding to 48 FliM units per flagella [40] (a value close to the one found in enterobacteria [41]), are probably located in the center of the C-ring, as proposed by a previous study using a cryo-electron tomography experiments on *L*. *interrogans* basal bodies [35]. It suggests an essential role of FliM in the assembly of the motor and probably of the whole flagella. Reduced amount of flagellar proteins and shortened flagella filaments may also be due to a defect of flagellar proteins export in absence of FliM. This may lead to protein degradation as they accumulate in the cytoplasm. Indeed, the C-ring is reported to form a thin wall surrounding the flagellar type III export apparatus used by flagellar proteins that have no leader sequences [38], like FlaB [2]. Other Sec-dependent secreted proteins like FlaA [2] may localize correctly but be degraded due to their inability to complex with other non-exported proteins like FlaB. In addition, it has been suggested that in *B*. *burgdorferi flgE* mutant and *B*. *hermsii fliH* mutant, FlaB, and possibly FlaA in *flgE* mutant, undergo post-transcriptional regulation beyond the simple protein degradation [37,42]. In a similar manner, loss of FliM may act as a negative regulator of flagellar proteins translation in *L*. *interrogans*. Structural characterization of the flagella by cryo-electron tomography as well as detailed protein-protein interaction experiments will be necessary to fully understand flagella assembly mechanisms in *Leptospira* and the critical role of FliM as scaffold protein for other flagellins such as FlaA and FlaB.

In summary, whole genome sequencing of a non-motile strain allowed the identification of a spontaneous point mutation which abolished FliM expression. This mutation in *fliM* resulted in destabilization of the flagella, leading to incomplete flagella assembly. Improper flagella formation affected motility and cell morphology. Moreover, thanks to the complementation study, motility was found to be directly linked to virulence. Further studies on the composition, protein interactions and structure of the leptospiral flagella should provide information about key mechanisms of the biology of these atypical bacteria and increase our understanding of the pathogenesis of leptospirosis.

## Supporting Information

S1 Fig Growth kinetics of 702^mot-^, WT and 702^compl^ strains. The 3 strains were inoculated at 1:100 onto 50 ml of EMJH and incubated at 29°C 80 rpm. The growth was followed by measuring the absorbance at 450 nm.
(TIF)

S2 Fig Motility of 702^mot-^, WT and 702^compl^ strains on 0.3% semi-solid medium. Spread of bacteria on soft 0.3% agar EMJH plates observed after 10 days of incubation.
(TIF)

S3 Fig Distribution of cell sizes of 702^mot-^, WT and 702^compl^ strains. 275 cells were measured for each strain on 3 different images taken at ×20 magnification.
(TIF)

S4 Fig Detection of FliM and LipL41 proteins in 702^mot-^, 702^compl^ and WT strains (uncropped gels). Cell lysates were performed in triplicates and analyzed by immunoblot to detect FliM (A) and LipL41 (B) proteins. Detection of LipL41 is used to confirm the equal loading of the lysates. The protein ladder, L1, is the MagicMark™ XP Western Protein Standard (Invitrogen).
(TIF)

S5 Fig Distribution of flagella sizes according to FlaB staining in 702^mot-^, WT and 702^compl^ strains. 210 flagella were measured for each strain on 10 different images taken at ×100 magnification.
(TIF)

S6 Fig Distribution of flagella sizes according to TEM observations in 702^mot-^, WT and 702^compl^ strains. 360 flagella were measured for each strain on 15 different images taken at ×4,800 magnification. Results from 3 independent batches of a same strain were pooled.
(TIF)

S7 Fig Flagellar protein content in purified flagella (PFs) and whole cell lysates in 702^mot-^, 702^compl^ and WT strains (uncropped gels). Whole protein contents of purified flagella, produced in triplicates, were revealed by Coomassie stained SDS-PAGE (A). Immunoblots of FlaA2 and FlaB (LIC11531) on purified flagella (B and C) and on whole cell lysates (D and E). The protein ladders, L1 and L2, are the Precision Plus Protein™ All Blue Prestained Protein Standard (Bio-rad) and MagicMark™ XP Western Protein Standard (Invitrogen).
(TIF)

S8 Fig Transcription of 8 genes likely involved in flagella assembly in 702^mot-^, WT and 702^compl^ strains. 8 genes involved in flagella assembly were chosen for a transcription assay, according to the annotations of their homologs in *L*. *interrogans* Copenhageni strain Fiocruz L1-130. These genes encode proteins of the filament (FlaA2, FlaB, FlaB4), the rod (FliE), the motor (FliG, FliN, FliF) and the flagellar type III secretion system (FliI). RT-PCRs were performed on mRNA previously normalized in quantity. *rpoB* is a housekeeping gene used as a control for normalization.
(TIF)

S1 Movie Supporting Movie 1. (AVI)

S2 Movie Supporting Movie 2. (AVI)

S3 Movie Supporting Movie 3. (AVI)
