# Supplementary figures and images for: Analysis of a Spontaneous Non-Motile and Avirulent Mutant Shows That FliM Is Required for Full Endoflagella Assembly in Leptospira interrogans
Source: PLoS One. 2016 Apr 4;11(4):e0152916. doi: 10.1371/journal.pone.0152916 (PMC4820103; doi:10.1371/journal.pone.0152916)

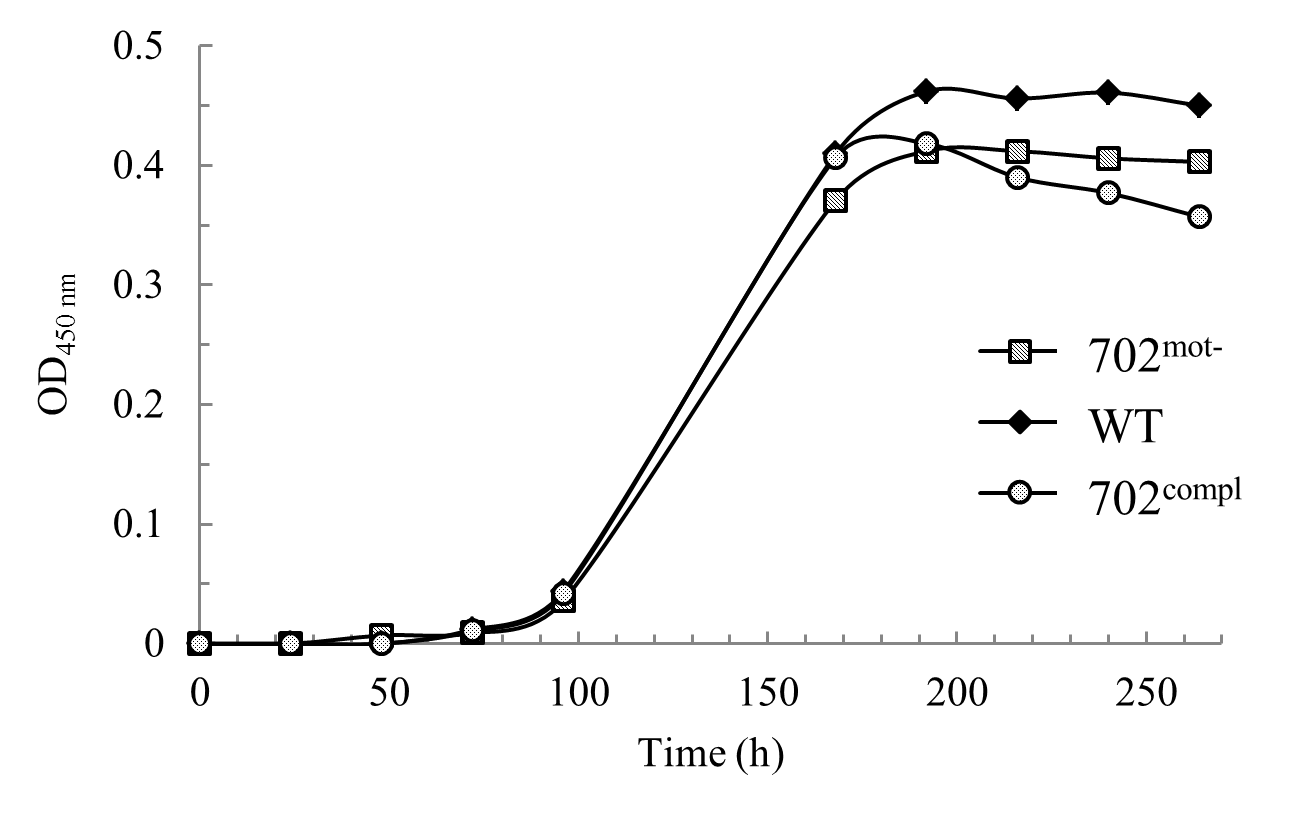

Supplement: S1 Fig — The 3 strains were inoculated at 1:100 onto 50 ml of EMJH and incubated at 29°C 80 rpm. The growth was followed by measuring the absorbance at 450 nm. (TIF) [file pone.0152916.s001.tif]

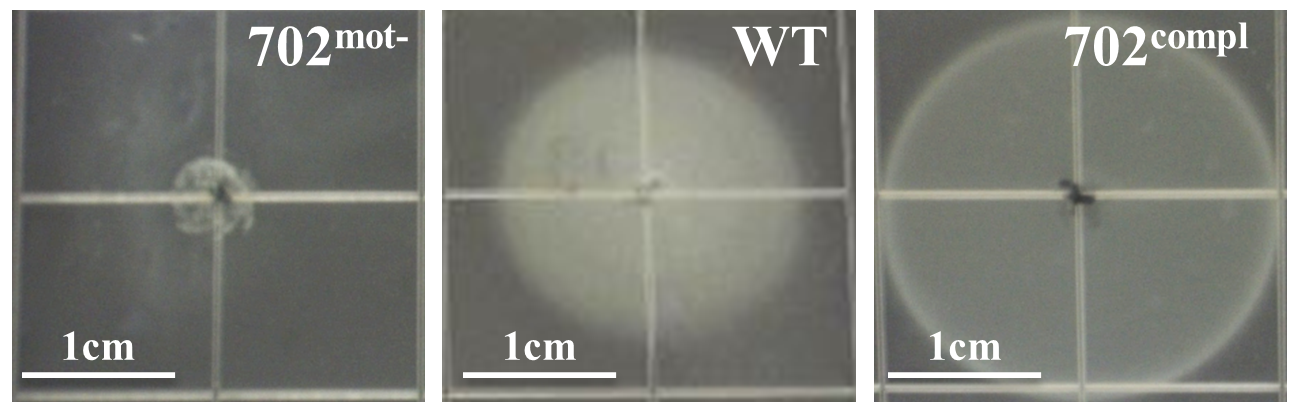

Supplement: S2 Fig — Spread of bacteria on soft 0.3% agar EMJH plates observed after 10 days of incubation. (TIF) [file pone.0152916.s002.tif]

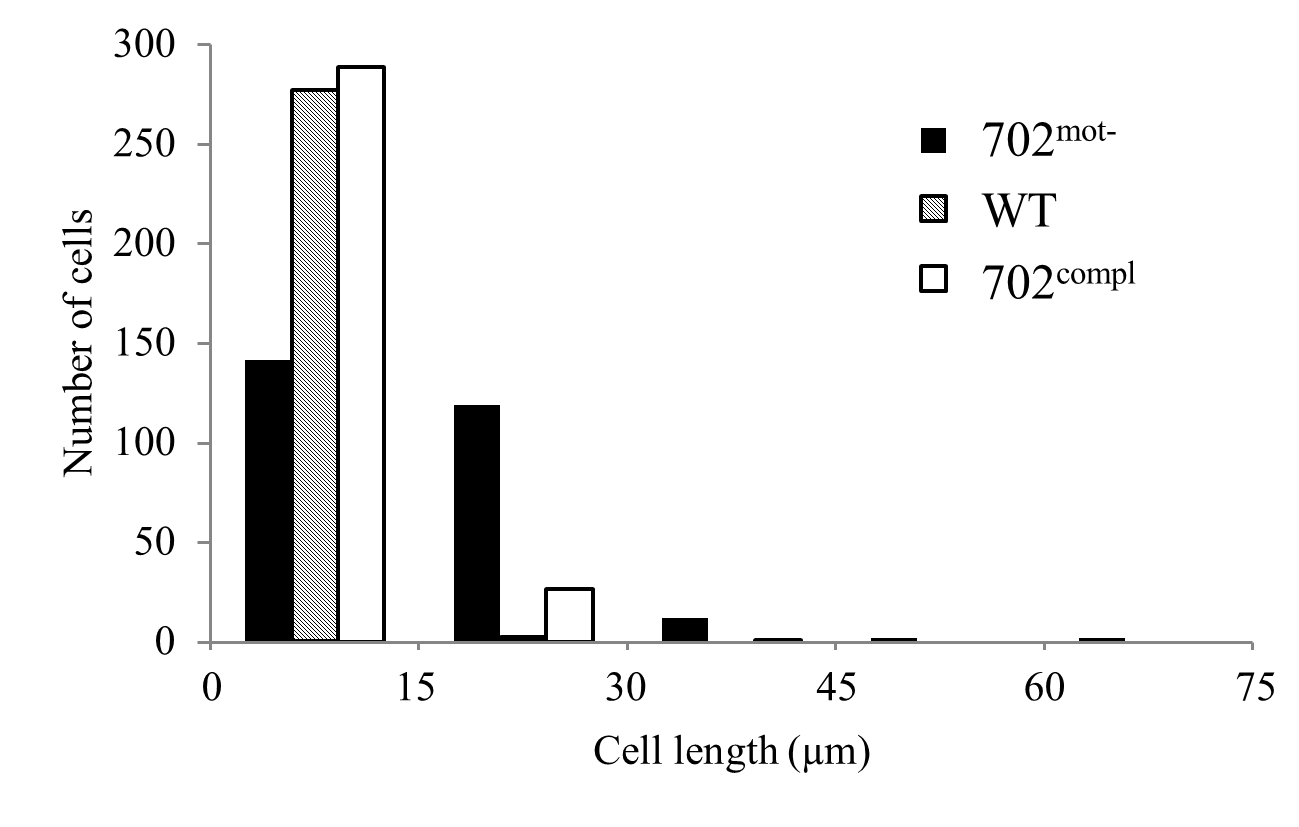

Supplement: S3 Fig — 275 cells were measured for each strain on 3 different images taken at ×20 magnification. (TIF) [file pone.0152916.s003.tif]

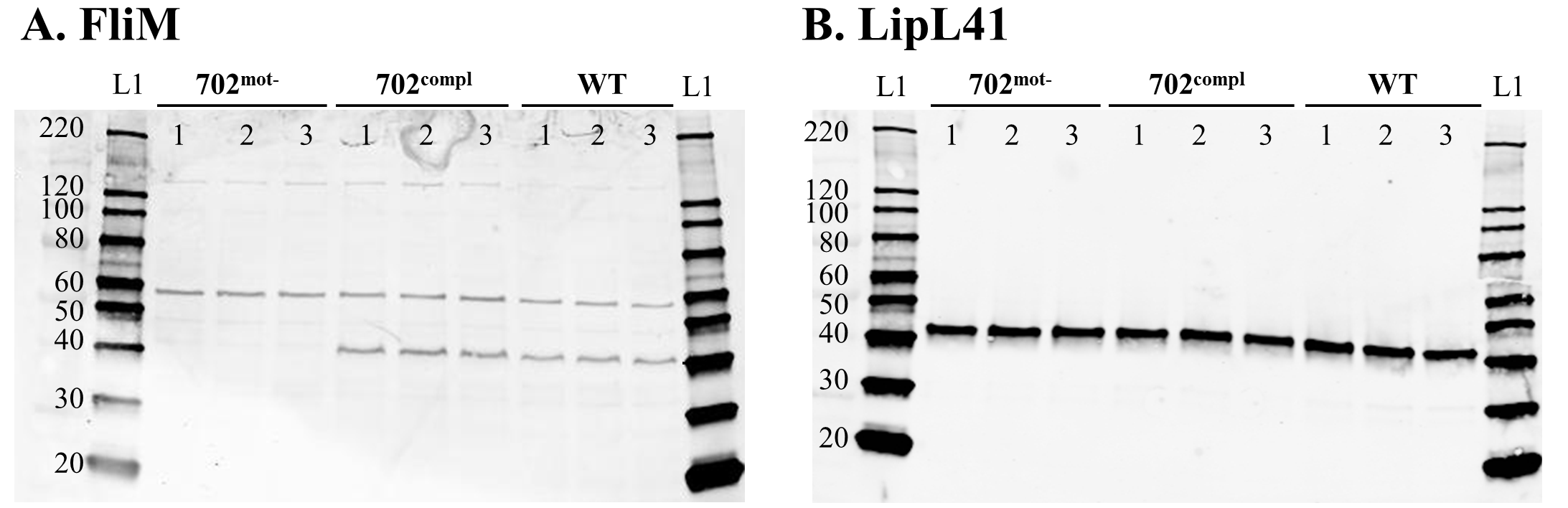

Supplement: S4 Fig — Cell lysates were performed in triplicates and analyzed by immunoblot to detect FliM (A) and LipL41 (B) proteins. Detection of LipL41 is used to confirm the equal loading of the lysates. The protein ladder, L1, is the MagicMark™ XP Western Protein Standard (Invitrogen). (TIF) [file pone.0152916.s004.tif]

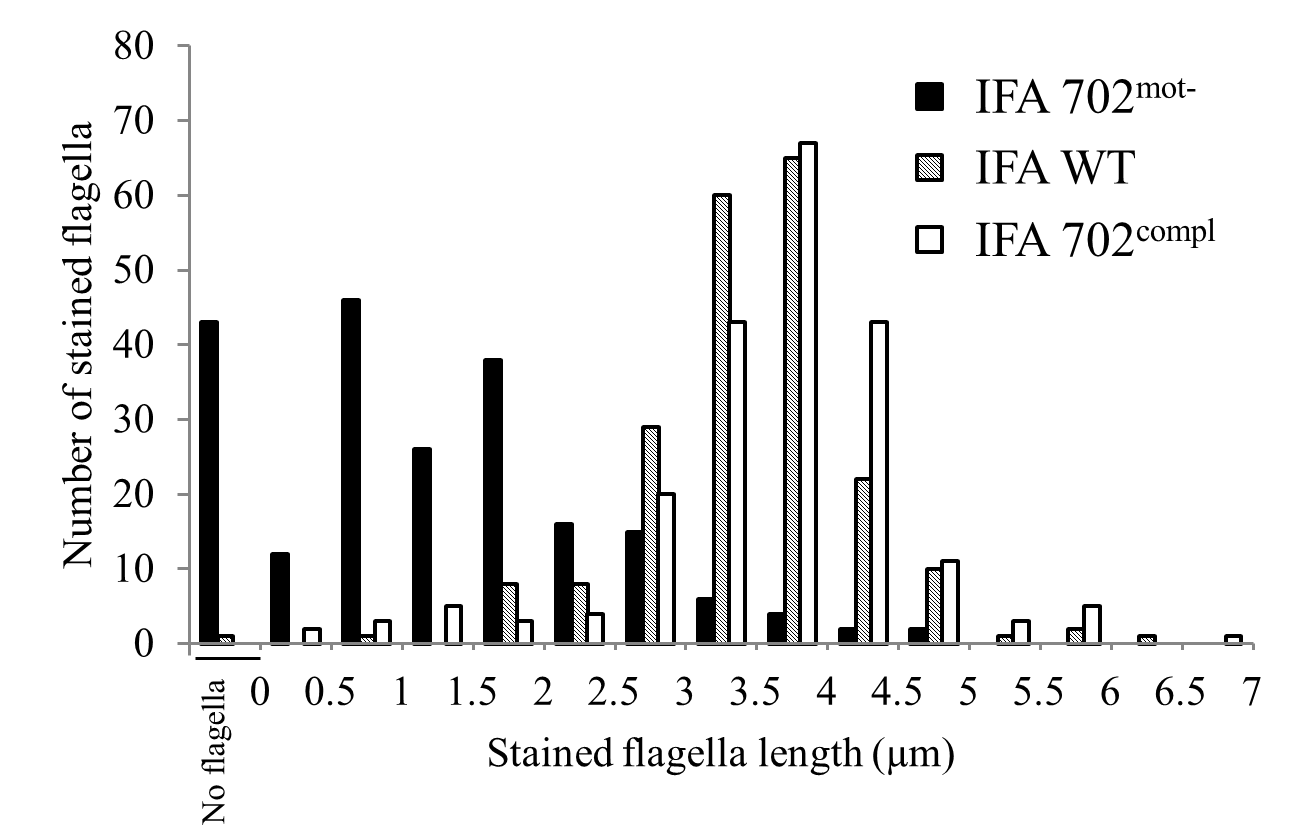

Supplement: S5 Fig — 210 flagella were measured for each strain on 10 different images taken at ×100 magnification. (TIF) [file pone.0152916.s005.tif]

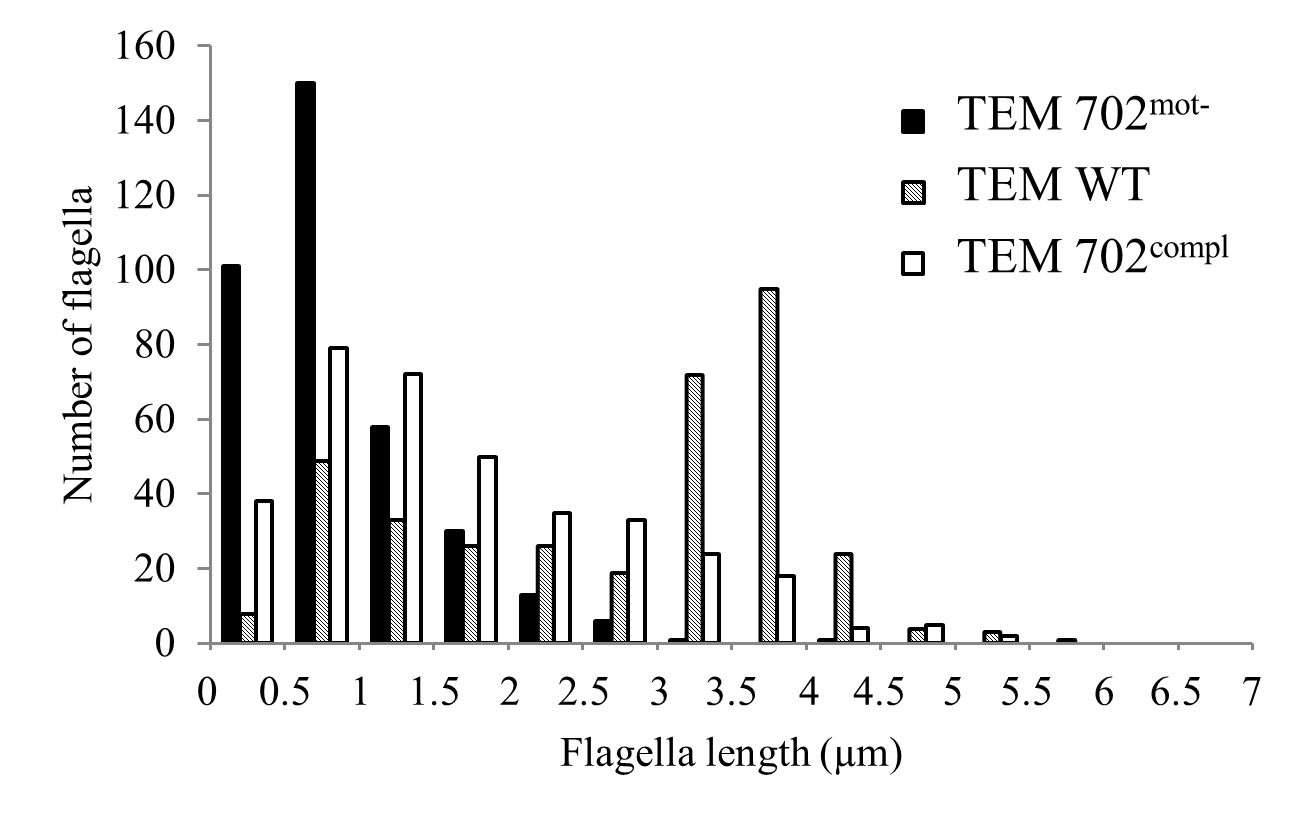

Supplement: S6 Fig — 360 flagella were measured for each strain on 15 different images taken at ×4,800 magnification. Results from 3 independent batches of a same strain were pooled. (TIF) [file pone.0152916.s006.tif]

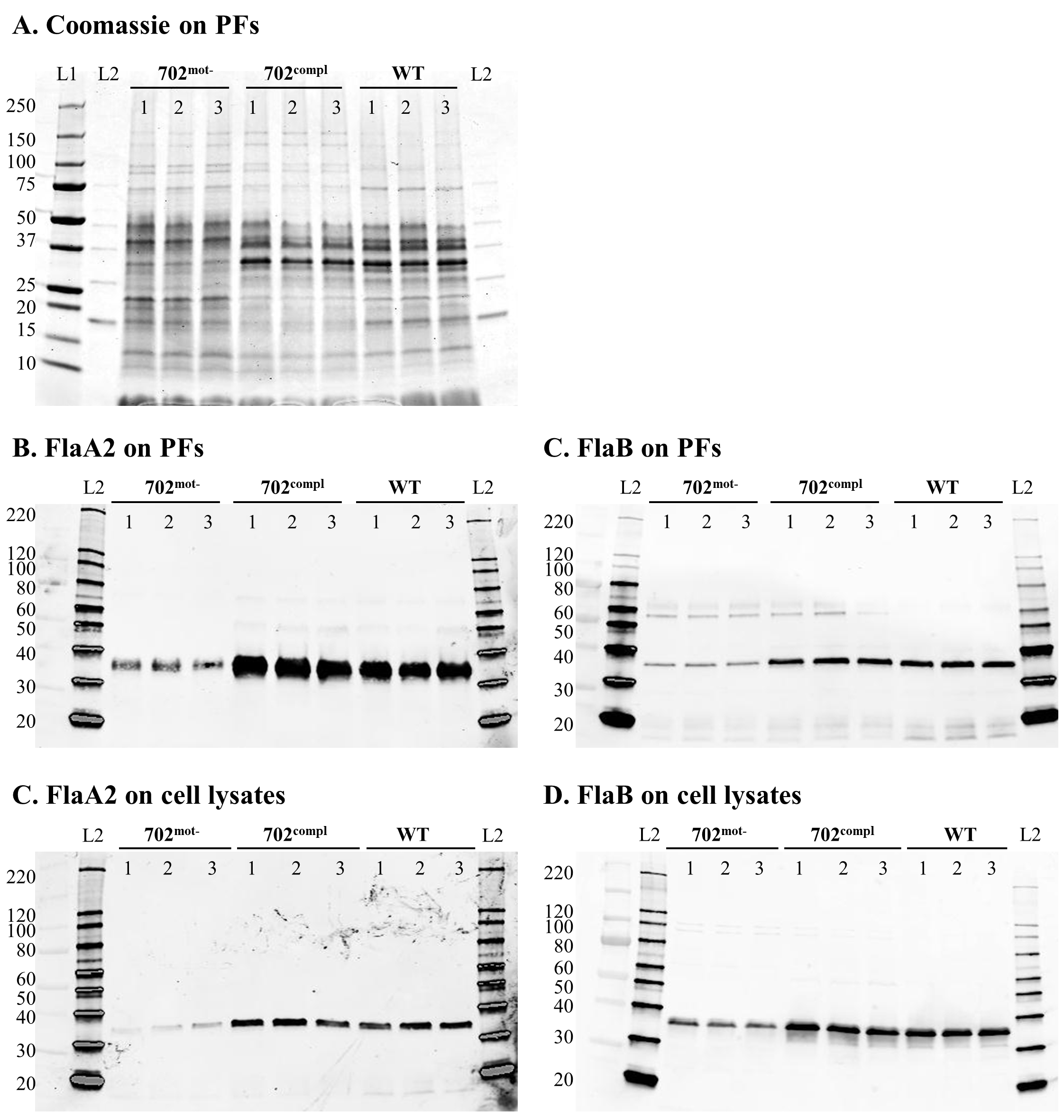

Supplement: S7 Fig — Whole protein contents of purified flagella, produced in triplicates, were revealed by Coomassie stained SDS-PAGE (A). Immunoblots of FlaA2 and FlaB (LIC11531) on purified flagella (B and C) and on whole cell lysates (D and E). The protein ladders, L1 and L2, are the Precision Plus Protein™ All Blue Prestained Protein Standard (Bio-rad) and MagicMark™ XP Western Protein Standard (Invitrogen). (TIF) [file pone.0152916.s007.tif]

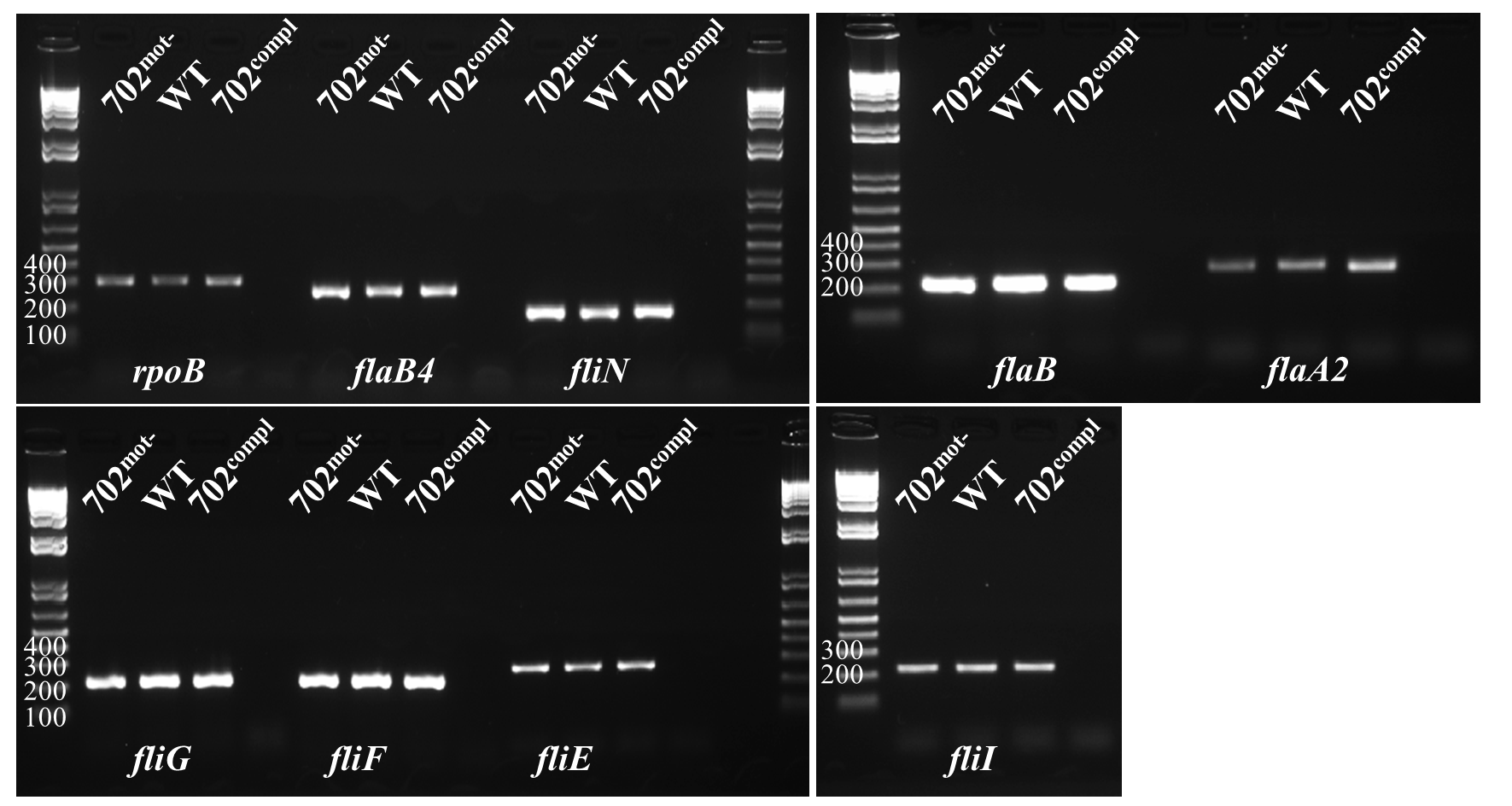

Supplement: S8 Fig — 8 genes involved in flagella assembly were chosen for a transcription assay, according to the annotations of their homologs in L. interrogans Copenhageni strain Fiocruz L1-130. These genes encode proteins of the filament (FlaA2, FlaB, FlaB4), the rod (FliE), the motor (FliG, FliN, FliF) and the flagellar type III secretion system (FliI). RT-PCRs were performed on mRNA previously normalized in quantity. rpoB is a housekeeping gene used as a control for normalization. (TIF) [file pone.0152916.s008.tif]
